# Management of lipedema with a biphasic ketogenic/low-carbohydrate diet: a case report

**DOI:** 10.3389/fnut.2026.1728651

**Published:** 2026-01-29

**Authors:** Roberto Cannataro, Diana Marisol Abrego-Guandique, Erika Cione

**Affiliations:** 1 Galascreen Laboratories, University of Calabria, Rende, Cosenza, Italy; 2 Department of Biology, University of Calabria, Rende, Cosenza, Italy; 3 Research Division, Dynamical Business and Science Society–DBSS International SAS, Bogotá, Colombia; 4 Department of Health Sciences, University of Magna Graecia Catanzaro, Catanzaro, Italy; 5 Department of Pharmacy, Health and Nutritional Sciences, University of Calabria, Rende, Cosenza, Italy

**Keywords:** inflammation, ketogenic diet, lipedema, low-carb diet, pain management

## Abstract

Lipedema is a multifactorial disorder with a negative evolutionary trend, influenced by genetic, hormonal, metabolic, and vascular factors that are not fully understood. Inflammation is a typical feature of lipedema and can be managed by limiting glycemic spikes. Herein, we report the case of a patient diagnosed with lipedema who followed a ketogenic diet (KD) for 6 months, resulting in a weight loss of 12 kg. Afterward, she transitioned to a low-carbohydrate (LC) diet for an additional 6 months, maintaining the good results achieved in terms of quality of life (QoL) improvement, weight control, and pain management. The patient regularly engaged in resistance training, which preserved and improved muscle mass. The primary and new outcome was due to the introduction of the second phase of the nutritional plan, specifically the low-carbohydrate diet, which could be an innovative approach. Often, diets that contain standard amounts of carbohydrates do not yield appreciable results.

## 1 Introduction

Lipedema is a condition affecting adipose tissue, first defined by Allen and Hines in 1940 (1). The diagnosis is clinical as neither biomarkers nor instrumental procedures are definitive, so its monitoring remains controversial (2). Lipedema primarily affects women, so it should be considered a gender-related condition (3, 4). Lipedema is a multifactorial disorder with a negative evolutionary trend, influenced by genetic, hormonal, metabolic, and vascular factors that are not fully understood. It is therefore reductive to define lipedema as an “abnormal fat deposition in the buttocks and bilateral legs, and can be accompanied by orthostatic edema,” [sic]as first postulated (1). Lipedema alters the physiology and likely the epigenetics of adipose tissue, rendering it non-responsive to conventional diets and physical activity. A genetic component is likely present, as hypothesized by Aksoy et al. (5) and partially confirmed by Paolacci et al. (6), implicating the *AKR1C1* gene. Generally, lipedema is triggered by hormonal changes. In addition to the genetic predisposition mentioned above, the pathophysiological process involving hormonal alterations is associated with estrogen receptors (ERs) in adipose tissue (7). Moreover, our group has recently outlined an epigenetic involvement (8), noting a different expression of selected microRNAs in adipose tissue affected by lipedema. It is worth noting that one of the pathways involved is related to the management of advanced glycation end-products (AGEs), molecules synthesized as a consequence of glycemic fluctuations. Microangiopathy is also present, resulting in minimal but constant tissue hypoxia and increased capillary permeability. This condition is responsible for transient edema, which is often aggravated by prolonged orthostasis, as well as a noticeable tendency to develop hematomas and petechiae, possibly related to an altered veno-arteriolar reflex (9, 10). At least 80–85% of patients report pain, either generally or after minor pressure. This pain could arise from tissue hypoxia associated with locoregional alterations in sensory nerve fibers or from tissue and fiber compression, thereby triggering an inflammatory response in the affected area (10). Lipedema is characterized by a bilateral and symmetrical increase in the volume of the affected limbs; only hands and feet are spared. Typical signs include the “handcuff” appearance at the wrists or the “sock” sign at the ankles; emblematic features include peri-rotular or perimalleolar fat pads. Bruises or petechiae develop rapidly in the affected areas, often after a slight bump or without a known cause. The skin appears fresh, pale blue, soft, and treatable; Stemmer’s sign is negative (7–10). Classification is divided into five types: i) localization at the buttocks; ii) localization at the buttocks and thighs; iii) localization at the buttocks, thighs, and entire legs; iv) localization limited to the arms; and v) localization limited to the calves. Generally, type V does not occur in isolation (7–10). Lipedema is further classified into four stages according to severity and appearance: i) stage 1: The skin appears smooth, and small nodularities are present, sometimes evident only during muscle contraction or upon compression of the affected area (7, 9). Stage 2: The skin exhibits a noticeable “peau d’orange” appearance, nodularities are also evident, and the subcutaneous tissue appears thickened. Stage 3: The skin appears “mattress-like,” nodules are larger, and there is a substantial disproportion between the affected and healthy areas. Stage 4: The lymphatic system is significantly affected to the extent that it is sometimes defined as lipo-lymphedema. A total of two variants are recognized: The nodular variant, in which fat accumulates in discrete pockets that may develop into macronodules in advanced stages; and the columnar variant, especially in the lower limbs, in which fat is distributed more uniformly, distorting the typical shape of the limbs. It should be emphasized that there is a significant phenotypic difference among individuals with lipedema, indicating that classification, diagnosis, and treatment are constantly evolving. Therapy should be based on teamwork, as there is no suitable drug or cure, but it can improve quality of life (QoL) and prevent disease progression. The main approach focuses on managing the inflammatory state. Patton et al. (11) reported, in a large sample, that more than one-third of individuals screened showed elevated C-reactive protein (CRP) levels. In this context, physiotherapy and elastic compression therapy are very useful (12). An appropriate nutritional approach is fundamental, even in the absence of overweight or obesity (13). The use of supplements is often recommended, although few have proven efficacy (14). Surgery is a valid option but is not wholly conclusive and should be considered alongside other treatments (15). The psychological approach also plays an important role in management (16). Finally, physical exercise aware of the inflammatory condition, should be a regular part of any program, preferably with resistance opposition. The main objective of conservative therapy for lipedema is to limit the inflammation characteristic of the disease. Traditional dietary schemes, providing 60–70% of total calories from carbohydrates, have shown poor or sometimes null results in achieving weight loss and improving clinical outcomes, particularly in terms of pain. For this reason, sometimes dietary schemes that tend to exclude foods or food groups, such as rare adipose disorder (RAD) diets or lactose-free diets, are considered. However, without a clear scientific rationale, there is no justification as to why these categories of foods should be avoided. In our experience (data not published), the prevalence of food intolerance is less than 20%. Similarly, the presence of HLA-DQ2 and HLA-DQ8 in some patients (17) does not imply that they will benefit from a gluten-free diet. Nevertheless, any clinically manifest intolerance must be taken into account, in which case the offending foods must be strictly limited (18). In our previous report, we showed how a ketogenic program that lasted for a very long time led not only to a weight loss of 41 kg but also a clear improvement in QoL and pain perception, without excluding any food (19). Similarly, Sørlie et al. (20) observed how nine women with lipedema experienced a significant reduction in pain after 9 weeks of a ketogenic diet (KD), but the benefits were lost upon returning to the previous diet. Jeziorek et al. (21) reported favorable results in terms of bodyweight, but they did not check QoL or pain. Di Rienzo et al. (22) reported an improvement in symptoms following a diet that included carbohydrates in a “Mediterranean-style” pattern. Therefore, a KD represents a valid tool, but it can also be considered a rational strategy for managing carbohydrate intake, given the potential hypersensitivity to glycemic fluctuations.

## 2 Case description

In this case report, we describe a journey lasting 12 months, where we first treated a patient with a ketogenic diet (KD) and subsequently with a low-carbohydrate (LC) diet, defined as a program with a carbohydrate intake of approximately 100 g per day. Obtaining results in terms of pain management and Quality of Life (QoL); so it could open a new scenario on management of lipedema, that it could rely not only on KD. The patient was a 41-year-old woman diagnosed with lipedema types IV and V, stages II-III. She complained of widespread pain, particularly in the lower limbs, as well as heaviness and difficulty performing various movements. She refused any treatment other than nutritional interventions. An assessment of pain and quality of life was conducted using the Western Ontario and McMaster Universities Arthritis Index (WOMAC) (23), the Sleep Quality Scale (SQS) (24), the RAND-36 (25), and the visual analog scale (VAS) (26) questionnaires. The KD is a nutritional program characterized by minimal carbohydrate intake (20–30 g per day or less than 5% of the total caloric intake). The ratio of the other two macronutrients can vary based on the objectives to be achieved. Under these conditions, the synthesis of ketone bodies is triggered, the more abundant is the β-hydroxy-butyrate (BHB), starting from liver fatty acids; these become the prevailing energy source. In this scenario there is normal glucose levels in blood due to the supply of gluconeogenesis. . It is now well established that, when implemented appropriately, this program has no contraindications (27). It has been used successfully in the management of migraines, in addition to its original application for epilepsy (28). Our group has used it to manage conditions characterized by pain, such as Tarlov cysts (29), as well as for polycystic ovary syndrome (30) and even cancer (31). In addition to being an effective program for weight loss, linked to an epigenetic action (32), it also shows anti-inflammatory properties, which are undoubtedly due to the absence of glycemic fluctuations but likely also result from a direct action of BHB. Another interesting mechanism is the regulation of antioxidant status, which is a consequence of reduced inflammation (33). The patient underwent regular resistance training, which preserved and improved muscle mass. This is a very important point, as it is reasonable to expect reduced muscle mass in lipedema. In fact, there is a shared document that underlines the importance of physical activity in the management of lipedema (34). In general, when considering a weight loss program, physical activity should be strongly recommended, especially to maintain long-term results (35). Under caloric restriction, it is likely that muscle mass could be negatively affected.

### 2.1 Bioimpedance analysis

Bioimpedance analysis (BIA) (36, 37) was performed using a bioimpedance analyzer (BIA 101 Anniversary, Akern, Florence, Italy), which employed a phase-sensitive device operating at 50 kHz. The accuracy of the BIA instrument was tested before each measurement, according to the manufacturer’s instructions. Measurements were conducted on a medical bed isolated from any electrical sources. The patient was in the prone position, with the legs (45° relative to the median line of the figure) and arms (30° from the chest) abducted. The skin was cleaned, and two electrodes were placed on the right hand and two on the right foot. Resistance (Rz) and reactance (Xc) were normalized to standing body height (m). The phase angle (PhA) was calculated as the arctangent of Xc/R × 180°/p; the LMI index was calculated as (Height x PhA)/Rz, providing a better indicator of muscle mass (38).

### 2.2 Nutritional biphasic plan

#### 2.2.1 Ketogenic phase

We chose to operate a caloric deficit of 200–250 kcal compared to the 14-day food diary reported, as we did in a previous study (19), to better design the nutritional program. Carbohydrate intake was set at no more than 25 g per day, and the ratio between proteins and fats ranged between 1: 1 and 1: 2. The decision to include a considerable protein intake aimed to preserve muscle mass as much as possible, which was already in suboptimal conditions, probably also due to lipedema. No kind of food was excluded; therefore, milk and dairy products, red meat, and even gluten were included (the latter in any case in small quantities, as it is related to carbohydrates). We considered nutritional supplements following our previously published guidelines (14): omega-3 fish oil (3 g per day of product, approximately 1.8 g of DHA + EPA) due to its proven anti-inflammatory action via resolvin mediators (39); vitamin C (1 g per day, divided into two doses) to compensate for possible deficiencies related to very low fruit intake and limited consumption of red or orange vegetable; and vitamin D (2000 IU per day), as we noted typical deficiencies in blood tests. All supplements were obtained from 4 + Nutrition, Padua, Italy. Table 1 shows an example of a daily meal. The patient also provided guidance and preferences for cooking, albeit with a few limitations; for example, even fried foods were allowed in moderation. After 2 months of the ketogenic program, we included a free meal with a carbohydrate content ranging from 60 to 120 g, which did not affect the program’s progress. The ketogenic program was maintained for 6 months before starting the LC diet.

**Table 1:** Example of the KD nutritional plan.

Nutritional plan	
Breakfast	100 g of seasonal fruit
	40 g of sliced turkey breast
	20 g of spreadable low-fat cheese
	1 espresso coffee without sugar or other caloric sweeteners
Snacks (during the day)	50 g of parmesan cheese
	20 g of nuts (walnuts, hazelnuts, cashews, almond)
	40 g of ham
	Vegetables ad libitum (from a list considering limited carbohydrate intake)
Lunch	200 g of low-fat ricotta cheese
	A large bowl of mixed salad
	1 tablespoon of extra virgin olive oil
	Vinegar and spices as desired
Dinner	1 whole egg and 100 mL of pasteurized egg white
	A large bowl of grilled vegetables (zucchini, eggplant)
	2 tablespoons of extra virgin olive oil
	Vinegar and spices as desired
Drink at least 2 L of water; carbonated sweetened beverages are also permitted	

#### 2.2.2 Low-carbohydrate phase

The second phase consisted of an LC diet, which allowed daily carbohydrate consumption. The program was structured with a daily intake of 90–100 g of carbohydrates, divided into at least two meal; however, we suggested that it could be consumed mainly in one meal, allowing the patient to include a substantial portion of pasta, rice, or bread (70–80 g), preferably whole-grain, and always eaten before a cup of vegetables to provide fiber and slow glycemic peaks. Other carbohydrates were obtained from fruits, preferably used as a snack. The amount of fat was lower than in the KD program to reduce overall calorie intake. Table 2 shows an example of a daily meal.

**Table 2:** Example of the low-carbohydrate nutritional plan.

Nutritional plan	
Breakfast	40 g of whole bread
	40 g of ham
	20 g of spreadable low-fat cheese
	1 cappuccino without sugar or other caloric sweeteners
Snacks (during the day)	150 g of seasonal fruit (only one time per day)
	20 g of nuts (walnuts, hazelnuts, cashews, almond)
	40 g of chicken sliced breast
	Vegetables ad libitum
Lunch	A large bowl of mixed salad
	70 g of whole pasta
	150 g of mixed seafood
	1 tablespoon of extra virgin olive oil
	Vinegar and spices as desired
Dinner	180 g of lean cut meat
	A large bowl of grilled vegetables (zucchini, eggplant)
	1 and 1/2 tablespoons of extra virgin olive oil
	Vinegar and spices as desired
Drink at least 2 L of water; carbonated sweetened beverages are also permitted	

## 3 Discussion

**Figure 1:**
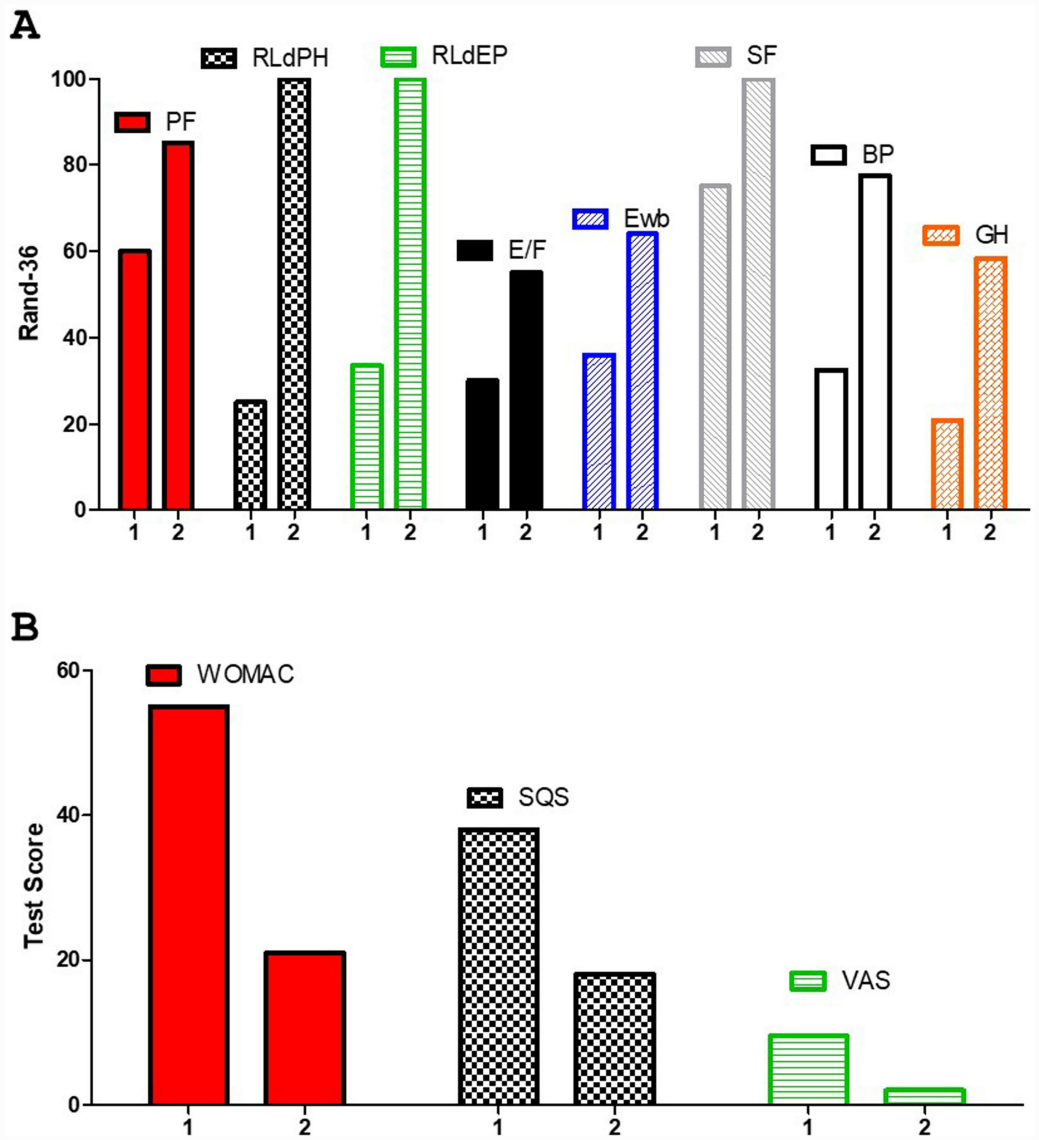
Health-related quality of life according to the RAND-36. **(A)** PF, physical functioning; RLdPH, role limitations due to physical problems; RLdPE, role limitations due to emotional problems; E/F, energy/fatigue; Ewb, emotional wellbeing; SF, social functioning; BP, body pain; GH, general health. **(B)** Western Ontario and McMaster Universities Arthritis Index (WOMAC), Sleep Quality Scale (SQS), and visual analog scale (VAS) questionnaire results collected at baseline (marked as 1) and at 12 months (marked as 2).

**Figure 2:**
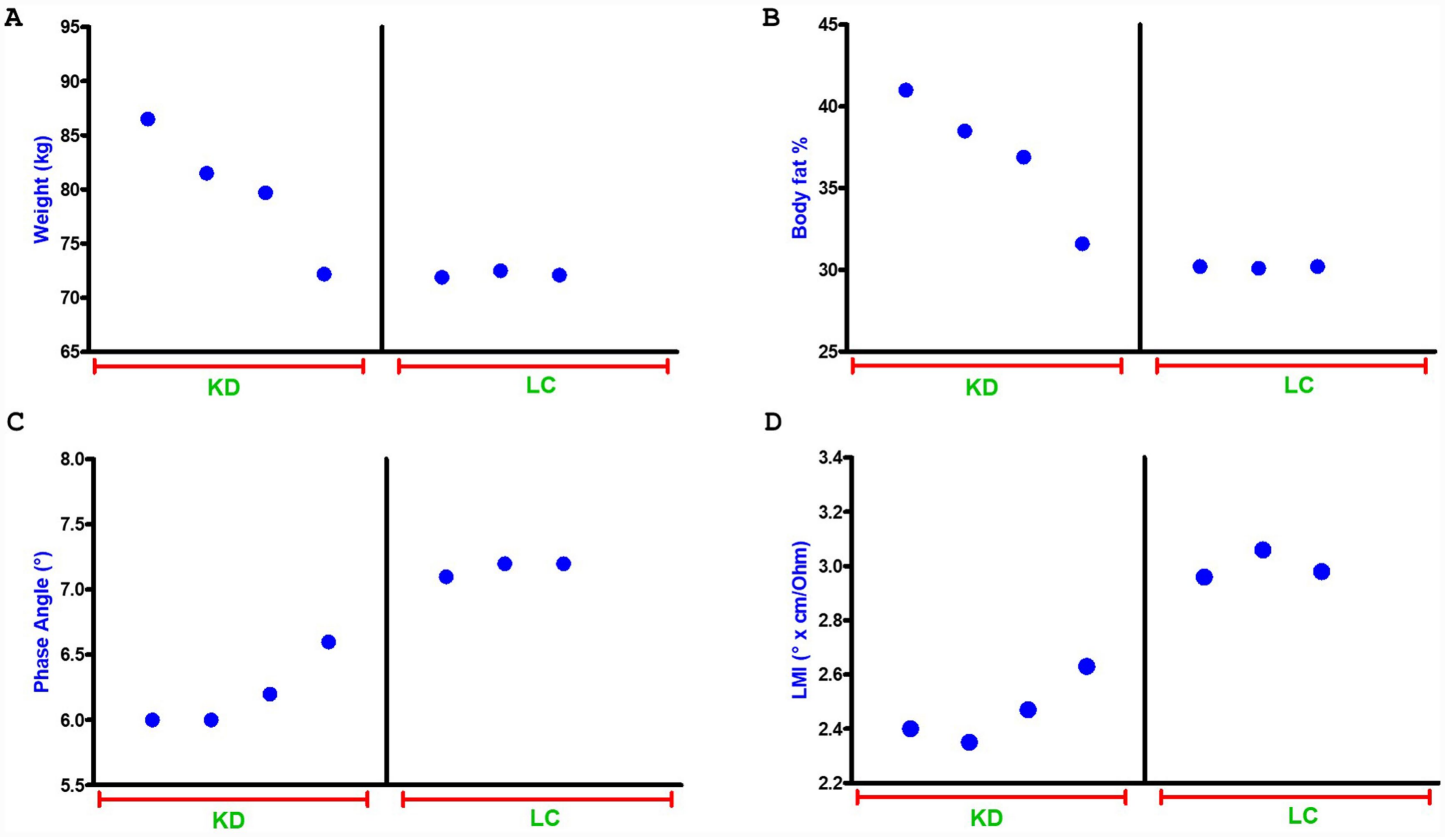
Anthropometric and bioimpedance parameters**. (A)** Weight; **(B)** body fat percentage; **(C)** phase angle; and **(D)** Levi’s muscle index (LMI) measured over 12 months during a biphasic diet combining a ketogenic diet (KD) and a low-carbohydrate (LC) diet.

The results confirmed the findings already reported in our previous study. Improvements in quality of life, assessed using the RAND-36 questionnaire, are shown in Figure 1A. Improvements in pain perception, measured using the WOMAC and VAS, as well as in sleep quality, were also noted, as shown in Figure 1B. Tests were collected at baseline (marked as 1) and after 12 months (marked as 2), as shown in Figures 1A,B. A decrease in body weight was observed, primarily due to a reduction in body fat, as directly demonstrated by BIA analysis (Figures 2A,B), and also reflected in the PhA and LMI values (Figures 2C,D). These parameters, being calculated from direct measurements rather than derived quantities or algorithm-based estimates, are considered more reliable. It is interesting to note that weight loss decreased significantly when carbohydrates were reintroduced (it should be noted that switching from a ketogenic diet to a low-carbohydrate diet typically results in at least 1 kg of weight gain due to the restoration of muscle glycogen and associated water (Figures 2A–D). However, with the introduction of physical activity, an improvement in indices related to muscle mass was observed. It is very likely that reintroducing carbohydrate enhanced the effectiveness of weight training, while the KD may have been less supportive of physical activity (40). Overall, improvements in quality of life were maintained. A weight training program using small weights and elastic bands, often performed at home, was supervised by a professional kinesiologist. Therefore, the program did not include a progressive increase in load but instead focused on maintaining the condition, with a greater increase during the LC phase. The WOMAC score decreased by more than 50%, as did the SQS. Direct measurement of pain was not possible due to the lack of pain biomarkers; however, the VAS indicated an overall improvement of 67%, approaching normality (Figure 2B). This could serve as an indirect verification of the KD’s anti-inflammatory effect. Of note, the effect of the KD on inflammation-related markers in humans was recently highlighted by a systematic review and meta-analysis of randomized controlled trials, which showed decreased levels of IL-6 after KD intervention (41). Moreover, in chronic rheumatic conditions (42) and in mechanically induced pain in animal models KD-LC diet are effectives (43). In addition, among 70 female patients with lipedema, LC appeared to be superior to a standard control diet in reducing pain, as well as body weight, body fat, and lower limb circumferences (21, 44, 45). As previously noted, the scientific literature on the lipoedema–nutrition link is limited. This case report serves as the starting point for designing larger trials, ideally involving a high number of participants following a dietary scheme that limits glycemic peaks without inducing ketosis. Such studies could include evaluation of inflammatory cytokines and/or CRP, direct assessment of pain rather than relying solely on questionnaires, and analysis of miRNAs, which are modulated by diet—particularly the ketogenic diet (46)—and which may be characteristic of lipedema. This could help monitor disease progression or even provide a potential diagnostic tool.

## 4 Conclusion

The primary and new outcome was due to the introduction of the second phase of the nutritional plan, namely the LC diet. The reintroduction of 100 g of carbohydrates per day—no more than 60 g per portion and always paired with at least 8–10 g of fiber—led to improvements in quality of life and pain management for our patient. This finding is particularly interesting and may have two possible explanations: One related to the regulation of glycemic peaks during the LC phase, and the other related to the exercise performed as part of the biphasic program. Similar effects have been observed in another pathophysiological condition (47).

## Data Availability

The datasets presented in this article are not readily available because of ethical and privacy restrictions. Requests to access the datasets should be directed to the corresponding author.
